# Advancing sustainable bioplastics: chemical and physical modification of starch films for enhanced thermal and barrier properties

**DOI:** 10.1039/d4ra04263h

**Published:** 2024-07-31

**Authors:** Pooja N., Shashank S., Bhisham Narayan Singh, Nirmal Mazumder

**Affiliations:** a Department of Biophysics, Manipal School of Life Sciences, Manipal Academy of Higher Education Manipal Karnataka 576104 India nirmal.mazumder@manipal.edu; b Department of Biotechnology, Manipal School of Life Sciences, Manipal Academy of Higher Education Manipal Karnataka 576104 India

## Abstract

This study addresses the urgent need for sustainable alternatives to conventional plastics by focusing on modification of thermoplastic starch (TPS) derived from renewable biomass sources. Despite TPS's biodegradability and cost advantages, its limitations in mechanical strength and water resistance prompted the investigation of physical and chemical modifications. Ultrasonication, autoclaving, and cross-linking with substances like citric acid and STMP (sodium trimetaphosphate)/STPP (sodium tripolyphosphate) were employed, with citric acid crosslinking standing out for its significant enhancement of transparency, especially beneficial for packaging applications. Film thickness varied with modification methods, with ultrasonicated films exhibiting thinner structures. Differential scanning calorimetry revealed insights into molecular interactions, with citric acid crosslinked film showing a substantial increase in thermal stability of the polymer at 164 °C, while moisture content analysis showed the impact of ultrasonication on reducing water absorption and citric acid crosslinking enhancing dimensional stability. Water vapor transmission rate data highlighted the effectiveness of ultrasonication in creating films with reduced permeability, and citric acid cross-linked films demonstrated potential for tailored water vapor barrier properties. Static water contact angle results indicated the hydrophobicity of films, with citric acid crosslinked films showing significantly more hydrophobic surfaces. The study also delved into water solubility, emphasizing the influence of depolymerization in ultrasonicated films and strengthened starch networks in crosslinked films, affecting their biodegradability. In conclusion, this comprehensive exploration demonstrates the feasibility of producing robust starch films with improved physicochemical properties through physical and chemical modifications, offering potential solutions in the quest for environmentally friendly alternatives to traditional plastics.

## Abstract 2

This study addresses the urgent need for sustainable alternatives to conventional plastics by focusing on modification of thermoplastic starch (TPS) derived from renewable biomass sources.

## Graphical abstract

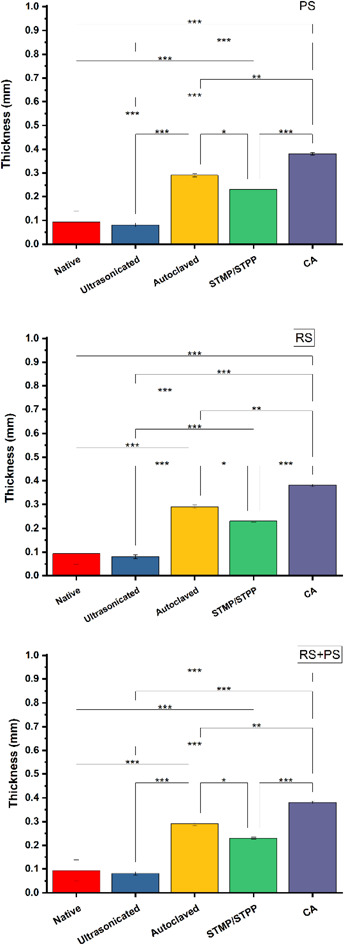

## Introduction

In recent times, there has been growing concern about the environmental impact of conventional plastics, which have been widely used for over a century and now dominate global material consumption. The overuse of plastics has led to significant harm to the environment, prompting the search for alternatives that are less harmful.^1^ Recycling rates for plastic waste remain low globally, emphasizing the urgent need for better waste management strategies.^2^ The packaging industry is a significant contributor to plastic waste, making it a critical area for implementing more sustainable practices.^3^ One promising alternative to traditional plastics is bioplastics, which are derived from renewable biomass sources such as rice, potato, corn, and vegetable waste.^4^ Bioplastics can possess both bio-based and biodegradable characteristics, making them attractive for various applications. Notably, they can degrade naturally in the environment, reducing the burden of plastic waste. Starch, a natural biodegradable polymer found in various plant sources, holds significant promise for bioplastic production.^5,6^ It is abundant, renewable, and relatively inexpensive, making it an attractive raw material. However, pure starch films have limitations in terms of water resistance and tensile strength, which hinder their widespread adoption.^7,8^ To overcome these limitations, researchers have explored various physical and chemical modifications to enhance the properties of starch-based bioplastics. Physical modifications such as ultrasonication and autoclaving can improve the physicochemical properties of starch films.^9,10^ Ultrasonication, defined as the application of sound waves above the normal human hearing range (anything above 20 kHz), is a method for physically modifying starch.^9^ This technique can be applied directly to gelatinized starch or to native starch granules suspended in a solution. Ultrasonication primarily targets the amorphous regions of the starch granules, causing physical degradation. The treatment results in porous surfaces and changes in physicochemical properties such as pasting properties, solubility, and swelling power. Factors such as temperature, treatment time, ultrasonication power, and frequency play critical roles in determining the behavior of the starch granules during ultrasonication.^11^ During ultrasonication treatment, gas bubbles form in the suspension medium, which bombard the starch granules before collapsing. This process, known as cavitation, creates shear forces that break the polymer chains, leading to polymer degradation.^12^ Additionally, solvent molecules dissociate to form radicals, contributing to further degradation. Studies have shown that sonicated starch granules exhibit an uneven surface and characteristic angular shapes, with some granules having visible cracks in the center.^13^ These structural changes can enhance the clarity of starch pastes, which is important for applications such as edible coatings and films. The degree of structural alteration in starch granules is influenced by factors such as temperature and treatment time. Autoclaving is another physical modification technique that involves subjecting starch to high pressure and temperature.^14^ This process disrupts the crystalline structure of starch granules, promoting the formation of more stable and uniform films. Autoclaving can improve the mechanical properties of starch films, such as tensile strength and elongation at break, making them more suitable for various applications.^15^ The combination of autoclaving and other modification techniques, such as ultrasonication and chemical cross-linking, can further enhance the performance of starch-based bioplastics. Chemical modifications of starch involve the use of cross-linking agents to form covalent bonds between starch molecules, resulting in improved mechanical strength and water resistance. STMP (sodium trimetaphosphate) and STPP (sodium tripolyphosphate) are commonly used cross-linking agents that can increase the stability and durability of starch films.^16–18^ These agents react with the hydroxyl groups of starch molecules, forming ester or ether linkages that enhance the film's properties. Citric acid, a naturally occurring organic acid, can also act as a cross-linking agent, improving the thermal and water stability of starch films.^19^ The combination of physical and chemical modifications can lead to significant improvements in the properties of starch-based bioplastics. This dual modification approach allows researchers to create bioplastics with a broader range of properties, suitable for diverse applications.^20^ Additionally, the incorporation of additives such as glycerol and sorbitol can plasticize starch, increasing its flexibility and processability without compromising its mechanical properties. The development of biodegradable membranes is another area of interest in the quest for sustainable alternatives to conventional plastics. Polymeric membranes are widely used for molecular separation processes in laboratories and industries, but traditional membranes are often made from non-biodegradable plastics. By developing biodegradable membranes, researchers aim to create more sustainable solutions for molecular separation processes. Biodegradable membranes can be made from natural polymers such as cellulose, chitosan, and alginate, as well as synthetic biodegradable polymers such as polylactic acid (PLA) and polycaprolactone (PCL).^19,21–24^ Additionally, the incorporation of additives such as plasticizers, fillers, and cross-linking agents can further enhance the properties of biodegradable membranes.^25–27^ Researchers are continuously exploring new formulations, processing techniques, and additives to optimize the performance of biodegradable membranes for various applications.

In conclusion, the development of bioplastics and biodegradable polymers offers a promising avenue to mitigate the environmental impact of plastic waste. Derived from renewable biomass sources, these materials can biodegrade naturally in the environment, reducing the burden of plastic waste. Various modifications, both physical and chemical, have been explored to enhance the properties of bioplastics, making them more competitive and sustainable alternatives to conventional plastics in diverse applications, including packaging, medical fields, and molecular separation processes. However, challenges remain in improving the mechanical and physical qualities of these materials and reducing production costs. Researchers are continuously exploring new formulations, processing techniques, and additives to optimize the performance of bioplastics and biodegradable polymers, making them more suitable for various applications and minimizing their environmental impact. Our study introduces a novel approach by combining different starch sources (PS and RS) and optimizing the crosslinking process to enhance the thermal and barrier properties of the resulting films. This dual modification strategy has not been extensively explored in previous studies, providing a new pathway for improving the performance of starch-based bioplastics. A-type starches, characterized by dense packing and high levels of rapidly digestible starch (RDS), readily break down during digestion. In contrast, B-type starches have less dense packing, allowing for increased levels of resistant starch, which resists enzymatic digestion and offers health benefits. By blending these two starch types and optimizing crosslinking processes, researchers enhance the thermal stability and barrier properties of resulting films. This dual modification strategy opens up new avenues for creating functional and sustainable materials.

## Experimental section

### Materials

Glycerol (extrapure AR, 99.5%), acetone, acetic acid (extrapure AR, ACS, ExiPlus, 99.9%), STMP (sodium trimetaphosphate), STPP (sodium tripolyphosphate), and sodium periodate were supplied by Sisco Research Laboratories Pvt. Ltd, India. Potato starch and rice starch were purchased from Sigma Aldrich, USA.

### Methods

Potato starch (PS), rice starch (RS), and a combination of potato and rice starch (RS + PS) were subjected to physical and chemical modifications and used to synthesize bioplastics.

### Physical modification

#### Autoclaved starch

25 g of starches (PS, RS, and RS + PS) were autoclaved at 120 °C for 1 h.^28^

#### Ultrasonicated starch

The process of ultrasonication was adapted from Sujka *et al.*^29^ 30 g of starch (PS, RS, and RS + PS) were dissolved in 100 mL of distilled water and were ultrasonicated for 30 min at 20 °C with a frequency of 20 kHz using a BioBee Tech ultrasonicator. The samples were cooled down by immersing in ice water. After centrifuging the samples, they were dried at room temperature for 24 h.

### Chemical modification

#### STMP/STPP crosslinked films

The protocol was adapted from Woggum *et al.*^16^ Take STMP and STPP in a 99 : 1 ratio, then add the STMP/STPP mixture to the beaker containing 18 g of starch dissolved in 62.5 mL of distilled water and stir the mixture. Using 5% NaOH, raise the pH to 10.5. When the pH reaches 10.5, place the beaker on a magnetic stirrer at 45 °C for 2 h. Using 1 M HCl, adjust the pH to 5.5 before washing the slurry with water. Centrifuge the tubes containing the mixture at 8000 rpm for 15 min. Allow the pellet to dry at 50 °C for 24 h after it has been formed.

#### Citric acid crosslinked films

50% (w/w of total starch weight) citric acid was allowed to react with starch at higher temperature close to 180 °C and stirred for 15 min.^17^

### Synthesis of starch films by solvent casting

The modified starch solutions were then used to produce starch films. The films were synthesized by solvent casting technique adapted from Amin *et al.*^26^ 3 batches of films were synthesized with 3 types of starch namely potato starch (PS), rice starch (RS), and a combination of the two starches in a 1 : 1 ratio (RS + PS). The films were made by dissolving 18 g of starch in 200 mL of distilled water in a beaker, then adding 8 mL of glycerol and acetic acid as plasticizers. The beaker was then placed on a hot plate and heated for about 45 min at 85–90 °C, stirring constantly. The molten starch mixture was carefully poured over the tray without leaving any air bubbles, and it was left at room temperature for 24 h before being placed to dry in a hot air oven maintained at 40–45 °C for 48 h.

### Characterization of synthesized bioplastics

#### Transparency

The transparency of the films was measured using a Varioskan LUX Multimode Microplate Reader. Transmittance, which indirectly indicates transparency, was determined by measuring the amount of light passing through the samples at different wavelengths (300–700 nm) and converting the absorbance to % transmittance using eqn (1).

1 % *T* = 10^(2 − absorbance)^

#### Film thickness

The thickness of the bioplastics was measured using a digital micrometer (Yuzuki, India) with a resolution of 0.01 mm. Measurements were taken at five different locations, and the average value was calculated.

#### Differential scanning calorimetry (DSC)

The thermal characteristics of the starch bioplastics were investigated using a DSC60 differential scanning calorimeter (Shimadzu Scientific Instruments, Japan). The films were conditioned in a desiccator over silica gel for 48 h to reduce moisture content. This conditioning process has been found to be effective in reducing the moisture to a minimal level, typically below 2–3%, which is generally considered acceptable for DSC analysis. The peak gelatinization temperature was determined by subjecting 2 mg of starch films with a heating ramp set to 5 °C min^−1^ from 30 to 200 °C. The transition temperatures were recorded at the onset of the peak. Moisture evaporation was minimized by preconditioning the samples in a desiccator prior to analysis.

#### Moisture content

The initial weights of 3 cm^2^ films stored at room temperature with 35% relative humidity for 48 h were recorded. The films were then oven-dried at 105 °C for 24 h, and the final weights were measured. The percentage of mass loss during drying was used to determine the moisture content of the bioplastics.^30^

#### Water vapor transmission rate (WVTR)

WVTR was determined by sealing circular films of 3 cm diameter on vials containing 30 mL of distilled water.^30^ The initial weight of the vials was recorded, and the setup was placed in a humidity chamber (Osworld Scientific Equipments Pvt. Ltd, India) for 24 h.^21^ Afterward, the vials were reweighed to determine the water vapor lost through the films, and WVTR (%) was calculated using eqn (2).

2 WVTR = Δ*W* × *A* × *t*

where, Δ*W* is the weight loss in grams, *A* denotes the cross-sectional area and *t* is time in h.

#### Static water contact angle

The hydrophobicity or hydrophilicity of the bioplastics was evaluated by measuring the static contact angle of water on their surfaces. A contact angle meter (Model No: HO-IAD-CAM-01; Holmarc Opto-mechatronics Ltd, India) was used to dispense a water drop on the different surfaces of the bioplastics, and the contact angle was measured after 10 seconds to maintain uniformity and consistency across all samples. The time frame ensures that any initial dynamic changes in the water drop shape are stabilized, providing a reliable and comparable measure of surface hydrophobicity.

#### Water solubility

To measure water solubility, 3 cm^2^ bioplastics were soaked in distilled water for 24 h with constant stirring. After removing surplus water with filter paper, the bioplastics were dried and their final weights were recorded.^30^ The % water solubility was calculated using eqn (3).3
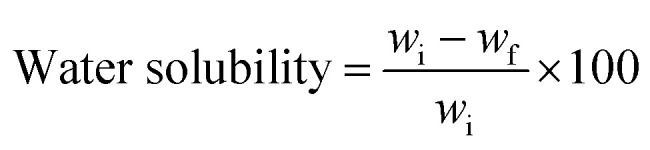


#### Biodegradation

The films were sliced into pieces measuring 2 cm^2^ and placed in pots filled with garden soil at a depth of 2 cm. This setup was kept in a greenhouse for a duration of 30 days. The weight of the elastomers was recorded every ten days following the soil burial. The biodegradability was assessed using eqn (4).

4 Weight loss (%) = [(*W*_o_ − *W*)/*W*_o_] × 100

where, *W*_o_ and *W* are the weights of samples before and after the test.

## Results and discussion

The synthesis of bioplastics from potato starch (PS), rice starch (RS), and a combination of both (RS + PS) involved a series of physical and chemical modifications to enhance the properties of the resulting films. Autoclaving the starches at 120 °C for 1 hour and ultrasonication improved the solubility and homogeneity of the starch, resulting in films with better flexibility. Chemical modifications with STMP/STPP and citric acid crosslinking significantly enhanced the thermal stability and water resistance of the films by forming strong covalent bonds between starch molecules. Both STMP and citric acid interact with starch hydroxyl groups, leading to crosslinking between starch molecules and the incorporation of citric acid moieties.

The chemical reaction between starch and STMP/STPP and citric acid is as follows:starch–OH + STMP → starch–O–STMP–O–starch + H_2_Ostarch–OH + citric acid → starch–O–citric acid–O–starch + H_2_O

The solvent casting technique ensured uniform thickness and minimized air bubbles, producing films that were transparent, flexible, and smooth. The RS films were more translucent and brittle, PS films were more opaque and more flexible, while RS + PS films showed intermediate properties. Crosslinked films exhibited superior durability, reduced water solubility, and maintained high biodegradability, making them suitable for environmental sustainability and potential applications in biodegradable packaging. These findings highlight the effectiveness of the modifications in tailoring the starch films' properties for specific industrial uses.

### Transparency

One of the important properties of bioplastics is transparency, which can impact their potential use in various applications, such as packaging materials. The study found that the transparency of the films was influenced by the amylose content of the starch. Amylose is a linear polymer found in starch that can form crystalline regions, affecting the light scattering within the material. Potato starch films, with lower amylose content, exhibited higher transparency compared to rice starch films. This finding is in line with previous research indicating that higher amylose content leads to decreased transparency in starch-based films. Moreover, the study investigated the effect of different modifications on film transparency. Among the various modifications, citric acid cross-linked films demonstrated the highest transparency. The incorporation of citric acid into the starch matrix might have influenced the molecular arrangement, reducing the scattering of light and resulting in higher transparency. This finding aligns with previous research, which suggests that higher amylose content leads to decreased transparency in starch-based films due to the formation of crystalline regions that scatter light (Table 1).^31,32^

**Table 1:** % Transmittance (normalized) of various starch films at different wavelengths. PS: potato starch; RS: rice starch; STMP: sodium trimetaphosphate; STPP: sodium tripolyphosphate; CA: citric acid

Sample		300 nm	400 nm	500 nm	600 nm	700 nm
PS	Native	0.0003	0.60	5.05	4.44	4.45
	Autoclaved	35.4	0.62	0.39	0.33	0.22
	Ultrasonicated	0.004	0.9	0.80	11.31	77.68
	STMP/STPP crosslinked	0.1	23.97	28.62	32.09	33.95
	CA crosslinked	1.26	36.24	43.68	46.90	48.57
RS	Native	0.000	0.48	1.12	4.13	3.70
	Autoclaved	55.69	0.10	0.04	0.03	0.01
	Ultrasonicated	0.031	1.23	1.63	2.76	5.76
	STMP/STPP crosslinked	0.08	10.92	15.07	17.02	19.15
	CA crosslinked	0.72	32.87	43.32	46.66	50.15
RS + PS	Native	0.01	5.50	6.01	8.68	8.68
	Autoclaved	58.56	6.24	4.14	3.80	3.10
	Ultrasonicated	0.20	5.14	4.13	11.64	24.23
	STMP/STPP crosslinked	0.0001	0.34	0.61	0.70	0.90
	CA crosslinked	1.37	39.55	50.35	55.47	59.05

### Film thickness

**Fig. 1:**
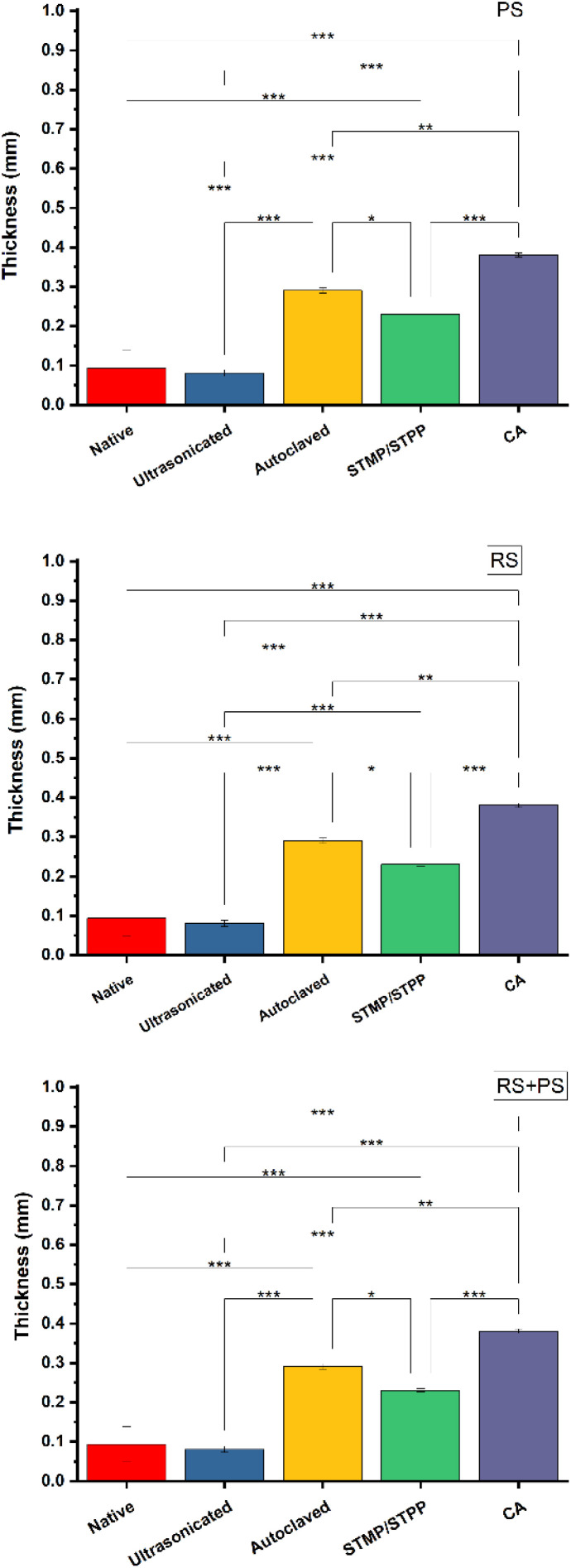
Thickness of different starch bioplastics. PS: potato starch; RS: rice starch; STMP: sodium trimetaphosphate; STPP: sodiumtripolyphosphate; CA: citric acid. *p ≤ 0.05 **p ≤ 0.01 ***p ≤ 0.001.

Film thickness is an essential parameter that can influence the mechanical and barrier properties of bioplastics. The study revealed that film thickness was determined by the viscosity of the film formulation. Ultrasonicated films were found to be thinner than the other starch films due to the improved tightness and compactness of the polymer matrix achieved during ultrasonication. This result suggests that ultrasonication could be an effective method to control film thickness, offering opportunities for tailored film properties.^33^ On the other hand, autoclaved films exhibited a thicker and more porous network structure. Autoclaving is a common method used to modify starch properties, and it can lead to the formation of a more open and porous structure due to the swelling and gelatinization of starch granules. This porous structure might have contributed to the increased film thickness. These findings are consistent with those reported in other studies, which have also demonstrated that processing methods significantly influence the morphology and properties of starch films (Fig. 1).^34^

### Differential scanning calorimetry (DSC)

**Fig. 2:**
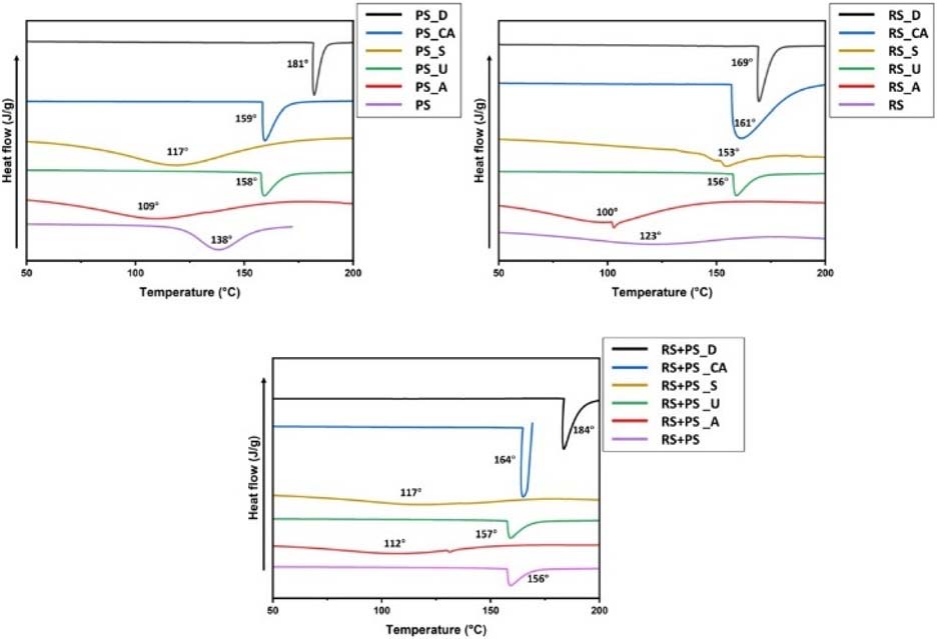
DSC endotherms of RS, PS and RS + PS films. PS: potato starch; RS: rice starch; S: STMP/STPP; U: ultrasonicated; A: autoclaved CA: citric acid.

DSC is a powerful technique used to investigate the thermal behavior of materials, including the gelatinization of starch. The study conducted DSC analysis on various starch films to assess their thermal characteristics. It was observed that the addition of citric acid increased the peak gelatinization temperature of the films. This finding suggests that citric acid cross-linking might have influenced the interaction between starch molecules, resulting in a higher gelatinization temperature. Gelatinization is a crucial process for starch-based materials because it affects their structural properties, such as crystallinity and molecular arrangement. By understanding the gelatinization behavior of the bioplastics, researchers can gain insights into their potential performance in applications where temperature changes might be involved. This finding aligns with other research, indicating that chemical modifications can alter the thermal properties of starch-based materials (Fig. 2).^35^

### Moisture content

**Fig. 3:**
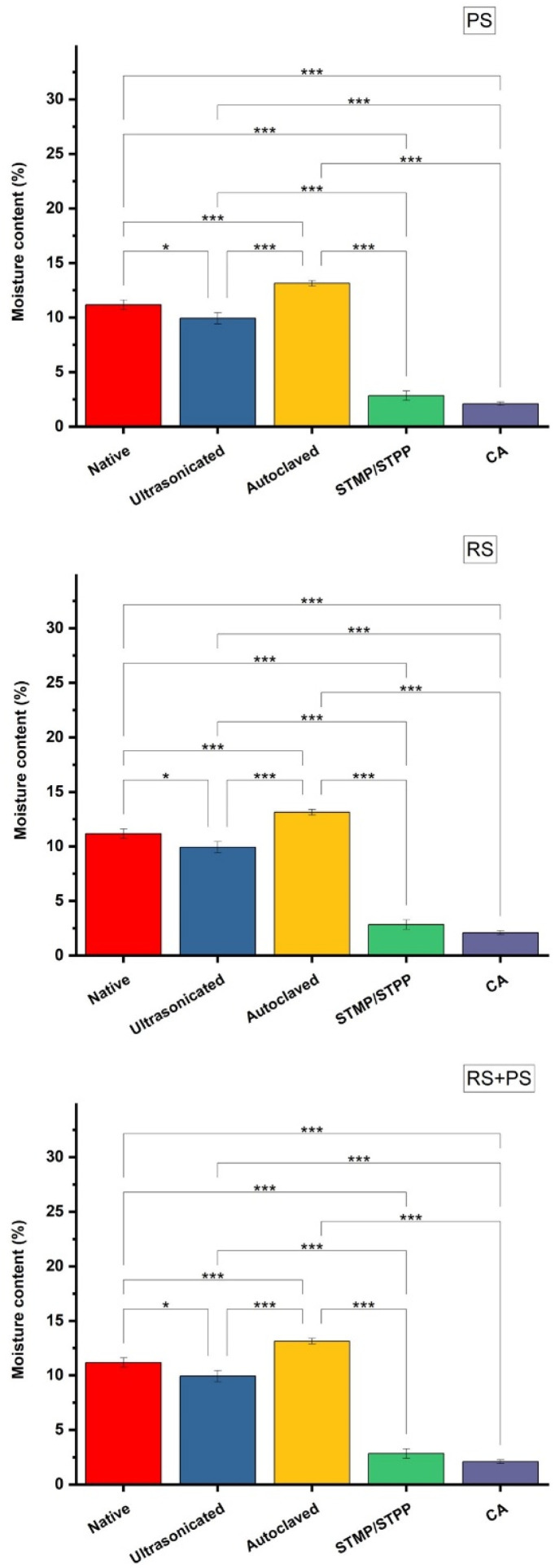
Moisture content (%) of different starch bioplastics. PS: potato starch; RS: rice starch; STMP: sodium trimetaphosphate; STPP: sodium tripolyphosphate; CA: citric acid. *p ≤ 0.05 **p ≤ 0.01 ***p ≤ 0.001.

Moisture content is an important property that can impact the stability and shelf life of bioplastic materials. The study investigated the moisture content of different starch films and found that ultrasonicated starch films had reduced moisture content compared to other films. Ultrasonication is known to cause depolymerization of starch, altering functional properties such as water solubility and moisture content. In contrast, citric acid cross-linked films showed the lowest moisture content among the various modified films. The presence of citric acid in the starch matrix could reduce the availability of hydroxyl groups, which are responsible for moisture absorption. This reduced moisture content is beneficial for bioplastics as it can improve their dimensional stability and reduce the potential for microbial growth. These observations are in line with previous studies, which have reported similar trends for chemically modified starch films (Fig. 3).^17^

### Water vapor transmission rate (WVTR)

**Fig. 4:**
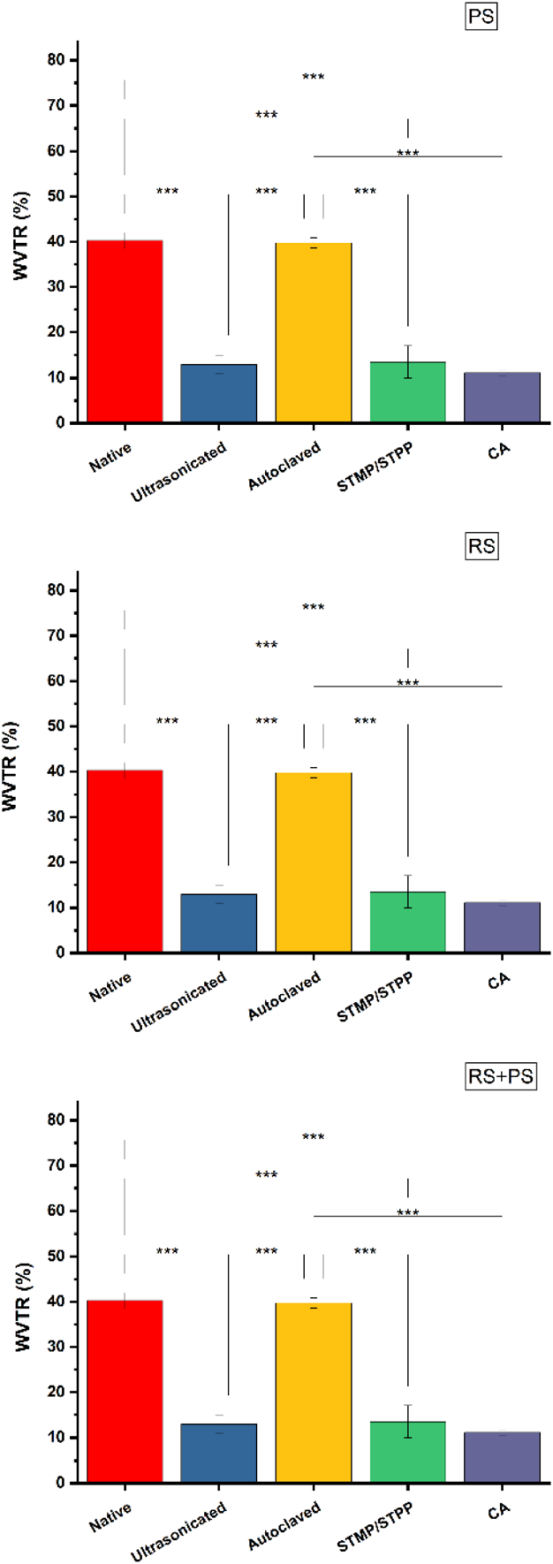
WVTR (%) of various starch films at 35 °C and 40% relative humidity. PS: potato starch; RS: rice starch; STMP: sodium trimetaphosphate; STPP: sodium tripolyphosphate; CA: citric acid. *p ≤ 0.05 **p ≤ 0.01 ***p ≤ 0.001.

WVTR is a critical property for bioplastics used in applications like food packaging, where the control of moisture transfer is crucial. The study found that ultrasonicated films had significantly reduced WVTR compared to other starch films. This could be attributed to the tightness and compactness of the polymer matrix achieved during ultrasonication, leading to a decreased rate of water vapor transmission. Additionally, citric acid cross-linked films also exhibited low WVTR. The cross-linking reactions in these films might have influenced swelling and molecular movement, resulting in a reduced diffusion coefficient and, subsequently, reduced WVTR. This finding suggests that citric acid cross-linking could be a promising approach to tailor the water vapor barrier properties of starch-based bioplastics (Fig. 4).

### Static water contact angle

**Fig. 5:**
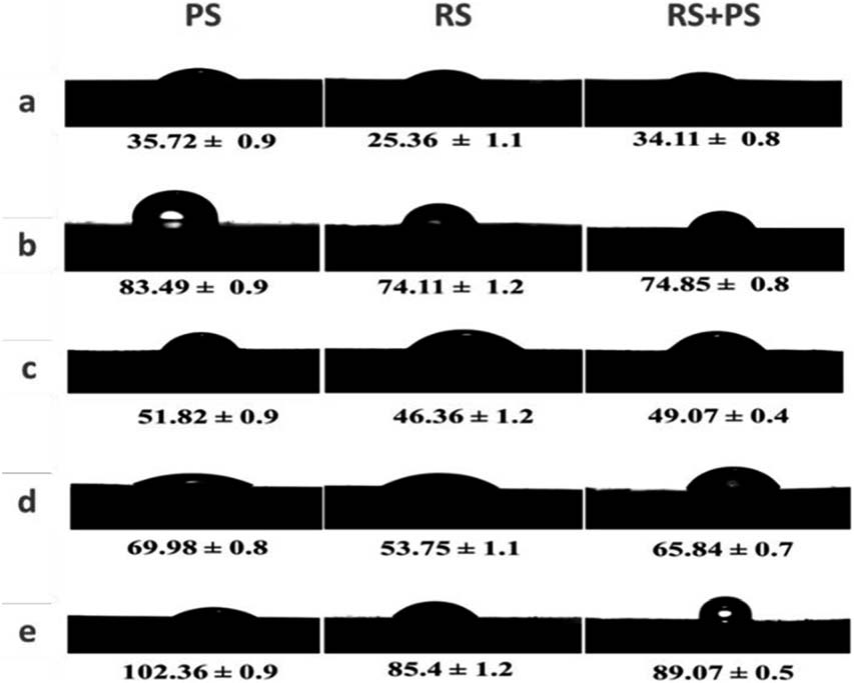
Static water contact angles of various starch films. (a) Native starch, (b) ultrasonicated starch, (c) autoclaved starch, (d) STMP/STPP crosslinked starch, and (e) citric acid crosslinked starch. PS: potato starch; RS: rice starch; STMP: sodium trimetaphosphate; STPP: sodium tripolyphosphate.

The hydrophobicity of the film surface was evaluated using water contact angles and is represented in Fig. 5. Hydrophobic surfaces have contact angles greater than 90 °C, while hydrophilic surfaces have angles less than 90 °C. Citric acid cross-linked films showed significantly lower water contact angles compared to other starch films, indicating a more hydrophobic surface. The hydrophobicity of bioplastics can be advantageous in applications where water repellence or resistance to moisture is desirable. The ability to control the surface properties of bioplastics through cross-linking could open up new possibilities for their use in various environments and applications.

### Water solubility

**Fig. 6:**
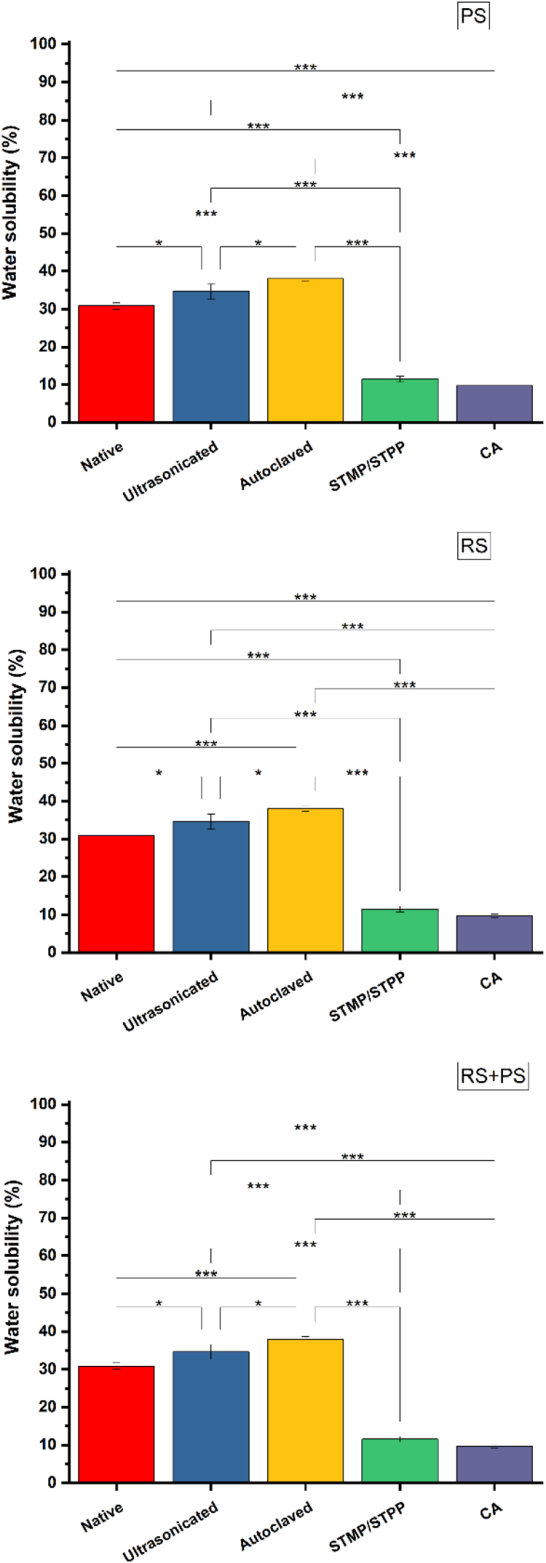
Water solubility (%) of different starch bioplastics. PS: potato starch; RS: rice starch; STMP: sodium trimetaphosphate; STPP: sodium tripolyphosphate; CA: citric acid. *p ≤ 0.05 **p ≤ 0.01 ***p ≤ 0.001.

Water solubility is a crucial property for biodegradable materials, as it determines their potential fate in the environment. The study evaluated the water solubility of different starch films and found that ultrasonicated films exhibited reduced water solubility due to depolymerization. On the other hand, cross-linking reactions, especially with citric acid, strengthened the starch network both chemically and physically, leading to decreased water absorption and increased water solubility. Understanding water solubility is vital for predicting the behavior of starch-based bioplastics in different environmental conditions, especially in scenarios where their potential for degradation is important. These observations are consistent with prior research that has investigated the impact of various modifications on the water solubility of starch films (Fig. 6).^36^

### Biodegradation

**Fig. 7:**
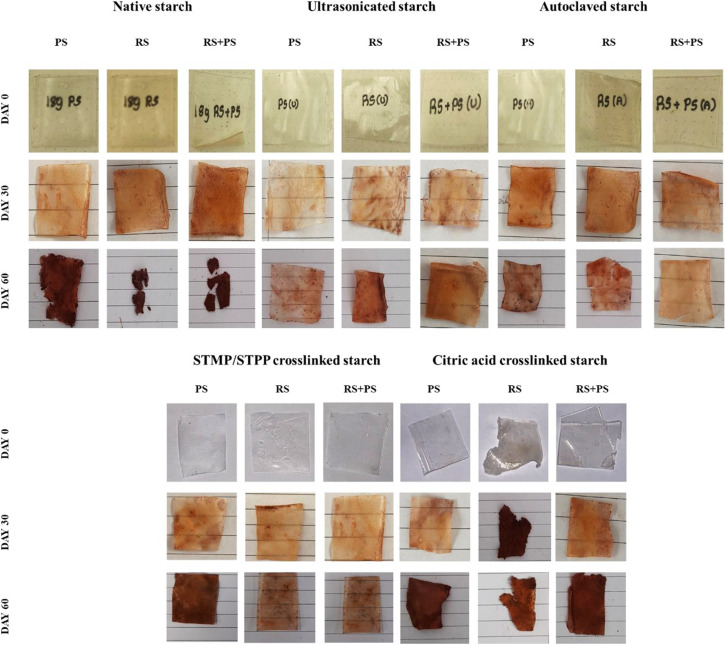
Photographs representing biodegradation of various starch films on days 0, 30 and 60. PS: potato starch; RS: rice starch; STMP: sodium trimetaphosphate; STPP: sodium tripolyphosphate.

The degradation behavior of various starch films has been investigated using the soil burial method, revealing distinct patterns for native starch films, ultrasonicated starch films, autoclaved starch films, STMP/STPP crosslinked starch films, and citric acid crosslinked starch films. Native starch films exhibited initial signs of degradation at day 30, reaching maximum degradation by day 60. Ultrasonicated starch films and autoclaved starch films demonstrated altered degradation kinetics compared to native films, indicating the impact of processing methods on their stability. STMP/STPP crosslinked starch films displayed enhanced resistance to degradation, suggesting the effectiveness of crosslinking agents in preserving film integrity. Citric acid crosslinked starch films exhibited slower rates of degradation, highlighting the potential of chemical modification to influence the material's stability. This comprehensive exploration of degradation characteristics provides valuable insights into tailoring starch films for specific applications through strategic modifications (Fig. 7).

## Conclusion

The poor degradability of conventional plastics has serious environmental consequences, including fossil fuel depletion and increasing pollution. Biopolymers, such as starch-based materials, play a crucial role in addressing these environmental challenges by reducing greenhouse gas emissions and offering biodegradable alternatives to traditional plastics. The packaging industry is a significant contributor to plastic waste, making it essential to adopt biopolymers with well-defined legislative guidelines to minimize environmental impact. Starch is one of the most abundant biopolymers and is widely used in the form of thermoplastic starch (TPS). However, TPS is known for its low tensile strength and water resistance, limiting its applicability. To enhance these properties, starch undergoes various modifications. Physical modifications, such as ultrasonication and autoclaving, can improve the physicochemical properties of starch films, leading to increased transparency, moisture content, water vapor transmission rate (WVTR), and hydrophobicity (water resistance). On the other hand, chemical modifications involve cross-linking starch with compounds like STMP/STPP and citric acid or oxidizing it with substances like sodium periodate and hydrogen peroxide. These chemical changes can enhance the hydration properties and overall performance of the developed films. The study focused on modifying native starch through both physical (ultrasonication and autoclaving) and chemical (crosslinking with STMP/STPP and citric acid) methods. Autoclaving increased the water solubility, moisture content, and hydration of the films, while ultrasonication improved transparency, moisture content, WVTR, and hydrophobicity. Notably, crosslinking with citric acid showed significant improvements in hydration and thermal properties of the starch films compared to other crosslinking methods. The comprehensive characterization of these synthesized bioplastics provided valuable insights into their properties, that can be enabled to create tailored bioplastics with specific characteristics for various applications, from food packaging to agricultural films. These efforts contribute to the ongoing global endeavour to develop sustainable alternatives to conventional plastics and mitigate their environmental impact.

## Data availability

The data supporting this article have been included in the main article and no new data were generated or analysed.

## Conflicts of interest

The authors declare no conflict of interest.

## References

[cit1] Patrício Silva A. L., Prata J. C., Walker T. R., Duarte A. C., Ouyang W., Barcelò D., Rocha-Santos T. (2021). Chem. Eng. J..

[cit2] Shafqat A., Tahir A., Mahmood A., Tabinda A. B., Yasar A., Pugazhendhi A. (2020). Biocatal. Agric. Biotechnol..

[cit3] Zimmermann W. (2020). Philos. Trans. R. Soc., A.

[cit4] Saharan B., Sharma D. (2015). Int. J. Microbiol. Res. Techno..

[cit5] Pooja N., Chakraborty I., Mal S. S., Bharath Prasad A. S., Mahato K. K., Mazumder N. (2024). RSC Adv..

[cit6] Maulida M. S., Tarigan P. (2016). J. Phys.: Conf. Ser..

[cit7] Ojogbo E., Ogunsona E. O., Mekonnen T. H. (2020). Mater. Today Sustain..

[cit8] Ribba L. , Garcia N. L., D'Accorso N. and Goyanes S., Disadvantages of Starch-Based Materials, Feasible Alternatives in Order to Overcome These Limitations, Elsevier Inc., 2017

[cit9] BeMiller J. N. , Starch Food Struct. Funct. Appl. 2nd edn, 2018, pp. 223–253

[cit10] Zhu F. (2015). Trends Food Sci. Technol..

[cit11] Hu A., Li Y., Zheng J. (2019). Lwt.

[cit12] Kaur H., Gill B. S. (2019). Int. J. Biol. Macromol..

[cit13] Carmona-García R., Bello-Pérez L. A., Aguirre-Cruz A., Aparicio-Saguilán A., Hernández-Torres J., Alvarez-Ramirez J. (2016). Starch/Staerke.

[cit14] Kim N. H., Kim J. H., Lee S., Lee H., Yoon J. W., Wang R., Yoo S. H. (2010). Starch/Staerke.

[cit15] Ulyarti U., Lisani L., Surhaini S., Lumbanraja P., Satrio B., Supriyadi S., Nazarudin N. (2022). J. Food Sci. Technol..

[cit16] Woggum T., Sirivongpaisal P., Wittaya T. (2014). Int. J. Biol. Macromol..

[cit17] Chakraborty I., Pooja N., Banik S., Govindaraju I., Das K., Mal S. S., Zhuo G. Y., Rather M. A., Mandal M., Neog A., Biswas R., Managuli V., Datta A., Mahato K. K., Mazumder N. (2022). J. Appl. Polym. Sci..

[cit18] Wilpiszewska K., Antosik A. K., Zdanowicz M. (2019). J. Polym. Environ..

[cit19] Ibrahim N., Kahar M., Wahab A., Uylan D. N., Ismail H. (2017). BioResources.

[cit20] Ashogbon A. O. (2021). Starch/Staerke.

[cit21] Woodruff M. A., Hutmacher D. W. (2010). Prog. Polym. Sci..

[cit22] Souza P. M. S., Sommaggio L. R. D., Marin-Morales M. A., Morales A. R. (2020). Chemosphere.

[cit23] Perotto G., Ceseracciu L., Simonutti R., Paul U. C., Guzman-Puyol S., Tran T. N., Bayer I. S., Athanassiou A. (2018). Green Chem..

[cit24] Le Digabel F., Avérous L. (2006). Carbohydr. Polym..

[cit25] N P. , Banik S., Chakraborty I., Mal S. S., Mahato K. K., Srisungsitthisunti P. and Mazumder N., in Frontiers in Optics + Laser Science 2022 (FIO, LS), Technical Digest Series, 2023, JTu5A.8

[cit26] Amin M. R., Chowdhury M. A., Kowser M. A. (2019). Heliyon.

[cit27] Tang S., Zou P., Xiong H., Tang H. (2008). Carbohydr. Polym..

[cit28] Soler A., Velazquez G., Velazquez-Castillo R., Morales-Sanchez E., Osorio-Diaz P., Mendez-Montealvo G. (2020). Carbohydr. Res..

[cit29] Sujka M., Jamroz J. (2013). Food Hydrocolloids.

[cit30] Talukdar M., Nath O., Deb P. (2021). Appl. Surf. Sci..

[cit31] Cano A., Jiménez A., Cháfer M., Gónzalez C., Chiralt A. (2014). Carbohydr. Polym..

[cit32] Li Y., Wang F., Xu J., Wang T., Zhan J., Ma R., Tian Y. (2023). Food Hydrocolloids.

[cit33] Mali S., Grossmann M. V. E., García M. A., Martino M. N., Zaritzky N. E. (2006). J. Food Eng..

[cit34] Jiménez A., Fabra M. J., Talens P., Chiralt A. (2012). Food Bioprocess Technol..

[cit35] Yu L., Dean K., Li L. (2006). Prog. Polym. Sci..

[cit36] Wu H., Lei Y., Lu J., Zhu R., Xiao D., Jiao C., Xia R., Zhang Z., Shen G., Liu Y., Li S., Li M. (2019). Food Hydrocolloids.

